# Trend Analyses on Interventional Treatment of Atrial Fibrillation From 2016 to 2022: Insights From a Multicenter Hospital Database of Left Atrial Catheter Ablation Cases

**DOI:** 10.1002/clc.70280

**Published:** 2026-04-27

**Authors:** Sebastian König, Sven Hohenstein, Johannes Leiner, Anne Nitsche, Henning T. Baberg, Michael Wiedemann, Melchior Seyfarth, Armin Sause, Alexander Staudt, Christopher Reithmann, Carsten Wunderlich, Jürgen Tebbenjohanns, Dong‐In Shin, Frank Steinborn, Michael Niehaus, Kerstin Bode, Andreas Bollmann

**Affiliations:** ^1^ Department of Electrophysiology Heart Center Leipzig at University of Leipzig Leipzig Germany; ^2^ Helios Health Institute, Real World Evidence & Health Technology Assessment Berlin Germany; ^3^ Department of Cardiology and Nephrology Helios Hospital Berlin‐Buch Berlin Germany; ^4^ Department of Cardiology University Hospital Helios Wuppertal Wuppertal Germany; ^5^ Department of Cardiology and Angiology Helios Hospital Schwerin Schwerin Germany; ^6^ Department of Internal Medicine I Helios Hospital München West Munich Germany; ^7^ Department of Internal Medicine II Helios Hospital Pirna Pirna Germany; ^8^ Department of Cardiology Helios Hospital Hildesheim Hildesheim Germany; ^9^ Department of Cardiology and Angiology Helios Hospital Krefeld Krefeld Germany; ^10^ Department of Cardiology/Interventional Electrophysiology Helios Hospital Erfurt Erfurt Germany; ^11^ Department of Internal Medicine I Helios Hospital Gifhorn Gifhorn Germany

**Keywords:** administrative data, atrial fibrillation, COVID‐19, left atrial catheter ablation, trend analyses

## Abstract

**Background:**

Current real‐world data on the utilization of atrial fibrillation (AF) catheter ablation (CA) are scarce, as is information on the impact of the COVID‐19 pandemic on trends in interventional AF treatment. Aims of this study were to describe case characteristics and trends of CA management using a contemporary multicenter database.

**Methods:**

In this retrospective, cross‐sectional analysis, we investigated administrative data provided by 87 German hospitals from 01/01/2016 to 12/15/2022. Based on ICD‐10 and OPS codes, inpatient cases with a main or secondary discharge diagnosis of AF who underwent CA were extracted. Incidence‐rate ratios (IRR) for case numbers with 95% confidence intervals (CI) were calculated using negative binomial models. Trends based on regression analysis were adjusted for baseline variables.

**Results:**

Analyzing 29 144 CA cases (89.4% from high‐volume centers), a significant increase in case numbers was observed throughout the study period (IRR 1.05, 95% CI 1.03–1.07, *p* < 0.001). There was no sustained impact on the overall trend from the COVID‐19 pandemic, but a temporary drop in case numbers in 2020. Utilization of transesophageal echocardiography (OR 0.82, 95% CI 0.81–0.83, *p* < 0.001) and intensive care treatment declined (OR 0.92, 95% CI 0.89–0.94, *p* < 0.001) and there was a trend toward a reduced incidence of pericardial tamponade. The ratio of cryoablations to radiofrequency CA case numbers increased from 0.29 ± 0.06 in 2016 to 0.50 ± 0.07 in 2022.

**Conclusion:**

We observed an increase in AF CA case numbers over the study period without a sustained influence of the COVID‐19 pandemic on this long‐term trend. Reported adaptations in CA management deserve further attention.

## Abstract (highlights)

**What Is Already Known?:**

- Catheter ablation is a first line treatment for patients with atrial fibrillation (AF) in several clinical situations.
- Inpatient treatment numbers of patients with AF were reduced in the early phase of the COVID‐19 pandemic, but no data are available on subsequent hospitalization trends.

**What The Study Adds:**

- This study is the first multicenter real‐world investigation on long‐term trends in the interventional treatment of patients with atrial fibrillation and left atrial flutter, including data of the COVID‐19 pandemic period.
- Throughout the study period, case numbers for catheter ablation procedures increased in both high‐ and low‐volume centers.
- Despite an increase in the average age of patients undergoing catheter ablation, the proportion of patients being treated in an intensive care unit decreased and there was a trend towards a reduction of cardiac tamponade.
- A relevant shift within utilized catheter ablation modalities in favor of cryoablation was to be observed within the study period.
- Observed trends were not relevantly influenced by the COVID‐19 pandemic.

## Graphical abstract

Based on the retrospective analysis of a multicenter inpatient administrative database from Germany comprising 29 144 patient cases with atrial fibrillation who underwent left atrial catheter ablation, this study describes an ongoing trend toward increased utilization of interventional therapy that was not sustainably influenced by the COVID‐19 pandemic.

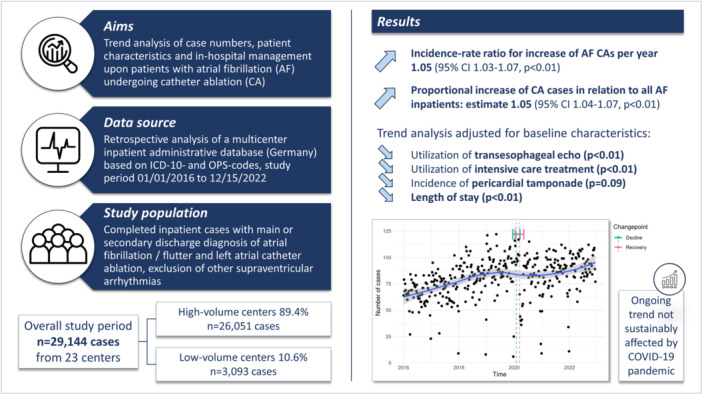

**Abbreviations**
AF: Atrial fibrillation
CA: Catheter ablation
CI: Confidence interval
COVID‐19: Coronavirus disease 2019
CrA: Cryoablation
ICD‐10‐GM: International Statistical Classification of Diseases and Related Health Problems [German Modification]
IRR: Incidence rate ratio
LoS: Length of stay
OPS: Operations and Procedures codes [German adaptation of the International Classification of the Procedures in Medicine of the World Health Organization, version 2017]
OR: Odds ratio
RFCA: Radiofrequency catheter ablation
TEE: Transesophageal echocardiography

## 1 Introduction

Left atrial catheter ablation (CA) for the treatment of atrial fibrillation (AF) was developed more than 25 years ago and has continued to evolve since then [1]. In recent years, adapted CA protocols resulting in optimized thermophysical conditions of lesion formation, technological developments related to existing ablation methods, and the introduction of new nonthermal CA techniques led to an improvement of CA therapy with streamlined yet clinically effective interventions and high levels of patients’ safety [2, 3, 4, 5]. In addition, current clinical studies have led to an expansion of indications for CA through the improvement of clinically relevant endpoints in certain patient groups [6, 7, 8]. As a consequence, there are guideline recommendations supporting CA as the most effective therapy for patients with AF in several clinical situations [9, 10]. However, up‐to‐date multicentric real‐world data on the implementation of CA and its subtypes into clinical practice is scarce especially for Europe and the evaluation of trends in CA utilization is mainly limited to studied periods before the onset of the Coronavirus disease 2019 (COVID‐19) pandemic. Especially in the early phase of the COVID‐19 pandemic, significant reductions in the number of in‐ and outpatients treated with cardiovascular diseases in general and AF in particular were noticeable [11, 12]. At the same time, a deficit of cardiovascular interventions and especially AF‐related CA procedures has been reported [13, 14]. There is a lack of data on whether these disruptive effects persisted in subsequent years. Therefore, the aims of this study were to [1] describe case numbers and case characteristics of patients who underwent a CA for AF over a period of 2016–2022 in a multicenter database [2], investigate trends in CA management and in‐hospital outcomes, and [3] assess the COVID‐19 pandemic's impact on the utilization of CA in Germany.

## 2 Methods

### 2.1 Case Selection

In this retrospective, cross‐sectional study, we investigated administrative data provided by 87 German hospitals from the Helios hospital network. All completed inpatient cases hospitalized between January 1, 2016 and December 15, 2022 were screened. Main and secondary diagnoses at hospital discharge according to the International Statistical Classification of Diseases and Related Health Problems (ICD‐10‐GM [German Modification]) as well as performed procedures encoded by Operations and Procedures codes (OPS [German adaptation of the International Classification of the Procedures in Medicine of the World Health Organization, version 2017]) were extracted from administrative data. Patient cases with a main or secondary diagnosis of AF or atrial flutter (ICD‐10‐GM: I48) and a concomitant OPS code for a left atrial CA (8‐835.23; 8‐835.25; 8‐835.33; 8‐835.35; 8‐835.43; 8‐835.45; 8‐835.83; 8‐835.9; 8‐835.a3; 8‐835.a5; 8‐835.b3; 8‐835.b5; 8‐835.c3; 8‐835.c5; 8‐835.d3; 8‐835.g3) were selected excluding those with an ICD‐10‐GM code for other supraventricular tachycardias (I47.1) or pre‐excitation syndrome (I45.6) at any position. Information on the proportion of radiofrequency‐based (8‐835.2ff, 8‐835.3ff) and cryoablation‐based (8‐835.aff) CA procedures as well as performed transesophageal echocardiography (TEE, OPS code: 3‐052) within the CA case were extracted based on OPS code groups. Comorbidities were structured based on a modified Elixhauser comorbidity index using the R comorbidity package [15]. The study period was divided into a pre‐pandemic (2016‐2019) and a pandemic interval (2020–2022) based on hospital admission date for subgroup analysis. Detailed information on used ICD‐10‐GM codes is provided in the Supporting Information S1: Table 1. Yearly case numbers were further categorized on a per‐hospital basis and only patient cases from hospitals that contributed at least one case per year and at minimum 10 cases per study period were used for the final analyses. High‐volume centers were defined by an average number of at least 75 AF CA cases per year within the pre‐pandemic period based on the requirements from the German Society of Cardiology [16]. Information on hospital admission type, treatment at an intensive care facility, length of stay (LoS), the occurrence of pericardial tamponade (OPS codes: 1‐842; 5‐370.0; 8‐152.0), and in‐hospital mortality were extracted from administrative data. In order to compare trends of CA utilization with trends in AF‐related hospitalizations overall, we identified all inpatient cases with a primary diagnosis of I48 at hospital discharge upon all Helios hospitals within the above‐mentioned study period without further procedure‐based selection criteria. There was no missing data. Data was stored in a double‐pseudonymized form and data use was approved by the local ethics committee of the University of Leipzig and the Helios Kliniken GmbH data protection authority. The study was conducted in accordance with the principles of good clinical practice and the Declaration of Helsinki. Considering the retrospective analysis of double‐pseudonymized administrative clinical routine data, individual informed consent was not obtained. The study follows the STROBE guidelines for cross‐sectional analyses.

**Table 1:** Yearly CA case numbers overall and stratified for center volume.

Year	CA case numbers		
	Overall cohort *n* = 29 144	Low‐volume centers *n* = 3093	High‐volume centers *n* = 26 051
2016	3364	349	3015
2017	3679	399	3280
2018	4233	434	3799
2019	4604	504	4100
2020	4261	440	3821
2021	4395	482	3913
2022	4608	485	4123

Abbreviation: CA, catheter ablation.

### 2.2 Statistical Analysis

Administrative data were extracted from QlikView (QlikTech, Radnor, Pennsylvania, USA). Inferential statistics were based on the R environment for statistical computing (version 4.0.2, 64‐bit build) [17]. For all tests, we applied a two‐tailed 5% error criterion for significance. Patient characteristics of the cohorts were described as numeric values, means with standard deviations or median with interquartile range. Numeric Differences in numeric variables were tested with linear regression. For the comparison of proportions of selected treatments and outcomes in the different cohorts, we used logistic regression and report proportions, odds ratios (OR) along with confidence intervals (CI) and *p* values. After testing for and confirming overdispersion in our data, case numbers were analyzed with negative binomial models [18]. We report incidence rate ratios (IRR, calculated by exponentiation of the regression coefficients) together with 95% CI comparing the two study periods, and *p* values. The analysis of LoS was performed via linear regression based on a transformation via inverse hyperbolic sine. We used linear regression to analyze the ratio between CrA and RFCA utilization, while fractional logistic regression was used for proportions of CA case numbers relative to case numbers with a main diagnosis of I48.

Analyses of trends of daily admissions were performed using Poisson regression. In addition to a linear term for day indices we used B‐splines of order 3 for (a) weekdays (1–7) and day index within year (0–1 for both regular and leap years). Trends for in‐hospital treatments were computed with and without adjustment for age, sex and Elixhauser comorbidity index. In order to investigate the impact of COVID‐19 pandemic on trends of CA case numbers, observed time‐series of weekly admissions were decomposed to an overall trend, a seasonal pattern, and a random part (Supporting Information S1: Figure 2). The detection of changepoints in the data was tested using segmented regression in both [1] the original time series and [2] the deseasonalized time series in order to detect changepoints that are not due to normal weekly patterns. We tested models with up to three changepoints and report results based on deseasonalized data only. The initial starting values for these changepoints were set to 150, 200, and 250 weeks. The selection of the optimal model was based on Akaike's Information Criterion.

## 3 Results

### 3.1 CA Case Numbers

**Figure 1:**
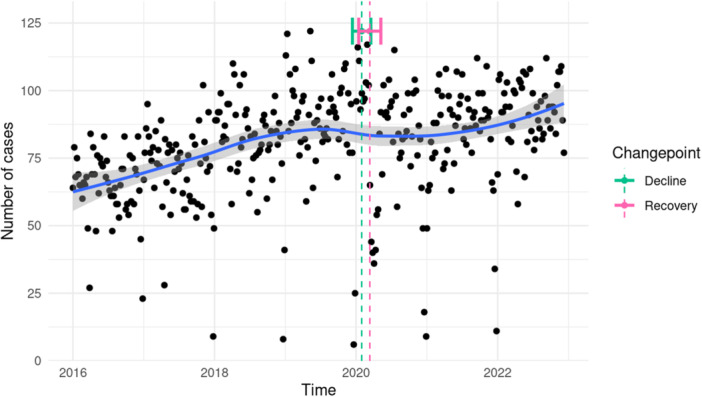
Temporal development of case numbers from the catheter ablation cohort. Based on weekly admission rates (smoothed curve). Shaded areas represent 95% confidence intervals. Error bars represent 95% confidence intervals for changepoints.

Overall, 29 144 CA cases from 23 centers were analyzed (Supporting Information S1: Figure 1), of whom 89.4% (n = 26 046) were treated in 12 high‐volume CA centers. The number of cases positively tested for the Severe acute respiratory syndrome coronavirus type 2 was negligible (*n* = 4 in 2020, *n* = 4 in 2021, *n* = 28 in 2022). Based on IRRs for daily admissions, there was a significant increase of CA case numbers over the years in the overall cohort (IRR 1.05, 95% CI 1.03–1.07, *p* < 0.001) and when separating high‐ (IRR 1.05, 95% CI 1.03–1.07, *p* < 0.001) from low‐volume centers (IRR 1.05, 95% CI 1.02–1.07, *p* < 0.001). Yearly case numbers are provided in Table 1, the development of CA admission numbers per week is illustrated in Figure 1. There was no significant effect of the study period on the trend of case admission numbers (pandemic vs. pre‐pandemic; *p* = 0.10) and no significant interaction between the study period and the general daily trend of increasing case numbers (*p* = 0.41) indicating no evidence for different trends. Based on time‐series analysis, the model that best described the development of CA case numbers (considering Akaike's Information Criterion) included two changepoints at the beginning of the COVID‐19 pandemic (Figure 1). After a dip in CA case numbers in the early phase, there was a quick recovery with a subsequent sustained and unchanged trend of increasing case numbers (Figure 1).

With regard to baseline characteristics in the overall cohort, mean age of CA cases increased over the years. There were no significant trends for changes in the distribution of sexes, total comorbidity burden or frailty risk. The trend of increasing mean age was driven by cases treated in high‐volume centers (average increase of years over time 0.35, 95% CI 0.29–0.41, *p* < 0.001), while no relevant trend was observed within cases contributed by low‐volume centers (average increase of years over time 0.10, 95% CI −0.08 to 0.28, *p* = 0.300). The absence of relevant time‐dependent changes in comorbidity burden was consistent across different types of hospital volume. Detailed baseline characteristics are provided in Table 2.

**Table 2:** Baseline characteristics stratified per year and trend analysis for the overall cohort of CA cases.

Variable	2016 *n* = 3364	2017 *n* = 3679	2018 *n* = 4233	2019 *n* = 4604	2020 *n* = 4261	2021 *n* = 4395	2022 *n* = 4608	Trend Estimate (95% CI)^a^	P for trend
Age [years]	64.3 ± 10.3	64.6 ± 10.4	64.8 ± 10.4	65.2 ± 10.5	66.2 ± 10.0	66.0 ± 10.3	66.0 ± 10.1	0.32 (0.26–0.38)	< 0.001
Age group ≤ 59 years [%]	29	30	30	29	24	26	25	0.95 (0.94–0.96)	< 0.001
Age group 60–69 years [%]	35	34	34	33	36	35	35	1.00 (0.99–1.02)	0.6
Age group 70–79 years [%]	33	33	33	33	34	33	34	1.01 (0.99–1.02)	0.4
Age group ≥ 80 years [%]	2.4	3.3	3.7	5.2	6.3	7.1	6.6	1.19 (1.16–1.22)	< 0.001
Female sex [%]	41	40	41	40	42	42	41	1.01 (0.99–1.02)	0.3
CHA_2_DS_2_‐VASc‐Score	2.3 ± 1.5	2.3 ± 1.6	2.3 ± 1.6	2.3 ± 1.6	2.4 ± 1.6	2.4 ± 1.6	2.3 ± 1.6	0.01 (0.00–0.02)	0.051
ECI	3.1 ± 7.1	3.4 ± 7.1	3.0 ± 6.9	2.9 ± 6.6	4.3 ± 6.8	3.5 ± 7.0	2.9 ± 6.5	0.00 (‐0.04 to 0.04)	> 0.9
ECI < 0 [%]	37	35	37	37	34	34	35	0.98 (0.97–0.99)	0.005
ECI = 0 [%]	19	19	20	19	19	20	23	1.03 (1.02–1.05)	< 0.001
ECI 1–4 [%]	8.4	9.4	9.2	9.3	10.0	8.5	8.3	0.99 (0.97–1.01)	0.4
ECI ≥ 5 [%]	35	37	34	35	37	37	33	1.00 (0.99–1.01)	0.7
HFRS	1.0 ± 1.8	1.0 ± 1.9	0.8 ± 1.8	0.8 ± 1.6	0.9 ± 1.8	1.0 ± 2.0	1.0 ± 1.7	0.01 (0.00–0.02)	0.14
HFRS < 5 [%]	96	95	97	97	97	96	97	1.02 (0.99–1.05)	0.3
HFRS 5–15 [%]	3.4	5.2	2.3	2.9	3.1	4.1	3.2	0.98 (0.95–1.02)	0.3
HFRS > 15 [%]	0.2	< 0.1	0.2	< 0.1	0.3	0.2	< 0.1	0.96 (0.83–1.11)	0.6

*Note*: Standard deviations (for continuous variables) and proportions are given in brackets within columns of yearly baseline characteristics.

Abbreviations: CA, Catheter ablation; CI, Confidence interval; ECI, Elixhauser comorbidity index; HFRS, Hospital frailty risk score.

^a^ Trends estimates for continuous variables represent an averaged absolute increase or decrease per year throughout the study period, trend estimated for proportions represent odds ratios for an increase ( > 1.0) or decrease ( < 1.0) over time.

### 3.2 In‐Hospital Care

Adjusted trends with regard to in‐hospital treatments revealed a decline in the utilization of intensive care therapy (averaged OR for per‐year comparison 0.92, 95% CI 0.89–0.94, *p* < 0.001) and TEE (averaged OR for per‐year comparison 0.82, 95% CI 0.81–0.83, *p* < 0.001) upon CA patients. These trends were consistent when differentiating cases from high‐ and low‐volume centers. There was a non‐significant adjusted trend for a decrease in the incidence of pericardial tamponade requiring intervention (averaged OR for per‐year comparison 0.94, 95% CI 0.87–1.01, *p* = 0.086) driven by a similar trend within high‐volume centers (averaged OR for per‐year comparison 0.92, 95% CI 0.84–1.00, *p* = 0.052), while in‐hospital mortality rates remained stable over time (averaged OR for per‐year comparison 1.06, 95% CI 0.77–1.47, *p* = 0.7) overall and in both hospital volume subgroups. There was a decrease in mean LoS (average difference in transformed variable −0.06, 95% CI −0.06 to −0.05, *p* < 0.001) both overall and in subgroups of high‐ and low‐volume centers with a longer averaged LoS in low‐volume centers (2016: 3.7 ± 3.0 days vs. 5.1 ± 4.8 days; 2022: 2.7 ± 2.6 days vs. 3.1 ± 2.7 days). Detailed results for trends in in‐hospital care utilization are provided in Table 3.

**Table 3:** In‐hospital care utilization within the overall cohort of CA cases.

Variable	2016 *n* = 3364	2017 *n* = 3679	2018 *n* = 4233	2019 *n* = 4604	2020 *n* = 4261	2021 *n* = 4395	2022 *n* = 4608	Trend Estimate (95% CI)^a^	P for trend
TEE [%]	90	81	63	62	61	63	52	0.83 (0.82–0.84)	< 0.001
Pericardial tamponade^b^ [%]	0.8	0.8	0.5	0.8	0.7	0.5	0.5	0.93 (0.87–1.00)	0.8
Intensive care therapy [%]	8.3	7.6	6.1	5.4	5.7	5.4	5.0	0.92 (0.89–0.94)	< 0.001
In‐hospital mortality [%]	< 0.1	< 0.1	< 0.1	0.0	< 0.1	0.0	< 0.1	1.03 (0.75–1.42)	0.8
LoS [days]	3.9 ± 3.2	3.8 ± 3.3	3.2 ± 2.9	3.2 ± 2.9	3.1 ± 3.0	3.1 ± 2.9	2.7 ± 2.6	−0.053 (−0.06 to −0.05)	< 0.001

*Note*: Standard deviations (for continuous variables) and proportions are given in brackets within columns of yearly baseline characteristics.

Abbreviations: CI, Confidence interval; LoS, Length of stay; TEE, transesophageal echocardiography.

^a^ Trends estimates for continuous variables represent an averaged absolute increase or decrease per year (of the transformed variable) throughout the study period, trend estimated for proportions represent odds ratios for an increase (> 1.0) or decrease (< 1.0) over time.

^b^ Pericardial tamponade requiring intervention (pericardial drainage or surgery).

Despite an increase in absolute numbers for both major CA techniques over time, the proportion of radiofrequency CAs (RFCA) upon all CA cases decreased (averaged OR for per‐year comparison 0.93, 95% CI 0.92–0.94, *p* < 0.001) while there was a relative increase in the utilization of cryoablations (CrA, averaged OR for per‐year comparison 1.06, 95% CI 1.04–1.07, *p* < 0.001). This trend was driven by a change in ablation technique utilization within high‐volume centers (RFCA: averaged OR for per‐year comparison 0.92, 95% CI 0.91–0.93, *p* < 0.001; CrA: averaged OR for per‐year comparison 1.08, 95% CI 1.06–1.09, *p* < 0.001), whereas an opposite trend for CrA utilization was observed in low‐volume centers (averaged OR for per‐year comparison 0.94, 95% CI 0.90–0.97, *p* < 0.001) despite the overall higher rate of CrA application (2022: 31% in high‐volume centers vs. 51% in low‐volume centers). The ratio of CrA to RFCA case numbers in the overall cohort increased from 0.29 ± 0.06 in 2016 to 0.50 ± 0.07 in 2022 with detailed results listed in Table 4.

**Table 4:** Ratios for CA/I48 case numbers and utilized CA techniques.

Variable	2016	2017	2018	2019	2020	2021	2022	Trend Estimate (95% CI)^a^	P for trend
Proportion CA/I48	0.17 ± 0.03	0.18 ± 0.03	0.20 ± 0.03	0.21 ± 0.04	0.21 ± 0.04	0.22 ± 0.04	0.22 ± 0.02	1.05 (1.04–1.07)	< 0.001
Ratio CrA/RFCA	0.29 ± 0.06	0.42 ± 0.10	0.47 ± 0.04	0.50 ± 0.05	0.47 ± 0.04	0.45 ± 0.05	0.50 ± 0.07	0.03 (0.02–0.03)	< 0.001

Abbreviations: CA, Catheter ablation; CrA, Cryoablation; RFCA, Radiofrequency catheter ablation.

^a^ Trend estimates represent odds ratios for an increase ( > 1.0) or decrease ( < 1.0) over time for proportion CA/I48 or an averaged absolute increase or decrease per year throughout the study period for the ratio CrA/RFCA.

### 3.3 Relationship Between CA Cases and Total Hospitalization Numbers of Patients With a Primary Discharge Diagnosis of AF or Atrial Flutter

**Figure 2:**
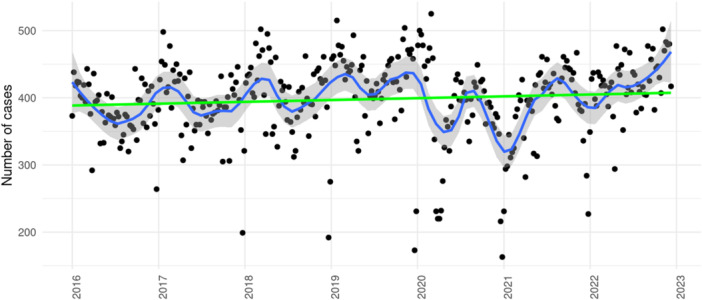
Temporal development of inpatient cases with a main discharge diagnosis of I48. Based on weekly admission rates. Shaded areas represent 95% confidence intervals.

**Figure 3:**
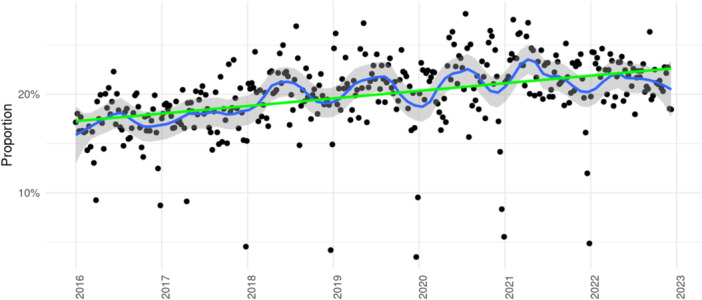
Temporal development of catheter ablation cases relative to inpatient cases with a main discharge diagnosis of I48. Based on weekly admission rates. Shaded areas represent 95% confidence intervals.

A total number of 144 449 inpatient cases with a primary discharge diagnosis of I48 was treated within the Helios hospital network between 2016 and 2022. Of them, 83 349 and 61 100 cases were hospitalized in the pre‐pandemic and the pandemic periods. Based on an IRR of 1.00 (95% CI 0.99–1.02, *p* = 0.489) for daily case admissions, treatment numbers remained stable over the years without a significant trend. Admission rates for I48 cases are illustrated in Figure 2. Calculating proportions from CA case numbers and case numbers with a main diagnosis of I48, a significant increase in the relative frequency of performing CA was to be observed (trend estimate 1.05, 95% CI 1.04–1.07, *p* < 0.001). Proportions (CA cases/I48 cases) ranged between 0.17 ± 0.03 in 2016 and 0.22 ± 0.02 in 2022 (Table 4), detailed results are illustrated in Figure 3.

## 4 Discussion

With this cross‐sectional study, we provide contemporary data on the evolution of case numbers of left atrial CAs for AF and left‐sided atrial flutter from a German multicentric clinical network. We observed an increase in case numbers for interventional AF therapy without evidence of a significant impact of the COVID‐19 pandemic on the overall trend throughout the study period. Modeling deseasonalized time‐series with changepoints within weekly admission numbers, we observed a dip with a subsequent sustained trend of increasing case numbers in the first half of 2020. Beyond that, there were changes in the in‐hospital management of CA cases with a decreased utilization of TEE and intensive care treatment in the observational period.

To date, no comparable data on AF CA case numbers up to the end of 2022 has been reported for Germany. A previous study examining in‐hospital treatment of patients with AF and atrial flutter in a multicenter German database reported of a trend toward the increased utilization of left atrial CAs for the period of 2010–2018 [19]. This trend appears to continue given the steady increase of case numbers. A more recent study reporting on a survey of German cardiology departments found a 38% increase in electrophysiological procedures, including a substantial proportion of AF CAs, when comparing case numbers from 2020 with those from 2015 [20]. Eckhardt and colleagues showed that the utilization of interventional therapy for AF steadily increased in Germany over a 10‐year period. However, their study was limited to data up to 2020 and did not explicitly investigate the impact of the COVID‐19 pandemic on the overall trend in detail [20]. Similar trends based on pre‐pandemic data were reported by single‐center as well as multicenter or even nationwide analyses from different countries [21, 22, 23, 24, 25]. There are multiple possible influencing factors, such as technological developments, the adaptation and streamlining of CA protocols, and the expansion of CA indications, as already outlined in the introduction section. Moreover, CA has been confirmed to be the most effective therapeutic strategy for AF in several clinical situations, which is likely to result in its earlier application within the treatment course of affected patients [26, 27, 28, 29]. Based on existing data, it may also be assumed that indications have been expanded and include AF patients with a more advanced disease and atrial myopathy [30].

Similar to the findings of our analysis, mean age of CA cases increased over time, as demonstrated in previous AF registries [22, 24]. Interestingly, other studies reported an increase of comorbidity prevalence, whereas we did not observe a relevant trend with respect to overall comorbidity burden. This could be explained by the examination of a comorbidity sum score in our study as compared to the prevalence analysis of specific comorbidities in other studies, with relevant differences reported for some, but not for all investigated variables [21, 24]. Nevertheless, it has been shown that CA is increasingly utilized in patient groups with certain relevant comorbidities such as chronic kidney disease [31]. The absence of relevant time‐dependent changes in the distribution of sexes within CA cases is in line with previous reports [25]. Regarding in‐hospital outcomes, a decline in the overall rate of procedure‐related adverse events including pericardial tamponades has been reported previously [22, 24, 30, 32]. We observed a corresponding non‐significant trend toward a reduction in the incidence of pericardial effusions requiring intervention that was driven by the cohort of CA cases treated in high‐volume centers. The declining proportion of intensive care treatment utilization within our data may also indicate a reduction in the occurrence of overall complications. Rates of incident relevant pericardial effusions and in‐hospital deaths were similar to comparable studies [33, 34].

We reported on a reduction of the overall LoS throughout the study period. Influencing factors to be considered include measures related to the COVID‐19 pandemic like adapted treatment pathways in order to reduce the risk of nosocomial infections. Irrespective of this, the significant reduction of performed TEEs starting in 2018 and the increased use of cryoablations indicate a general streamlining of processes related to CA interventions. There are no comparable multicenter data regarding the relative increase in cryoablation procedures, but the growing body of evidence with respect to its non‐inferiority compared to RF CA is likely to have an impact on this development. The implementation of modern procedural protocols as well as an increasing interventional experience have led to a reduction in procedure duration as shown in other trials [21, 23, 35]. This as well as the evolution of day‐care protocols related to AF CAs with proven safety and cost‐efficacy could have influenced inpatient management and the standard LoS following such interventions [36, 37].

Only a minority of the referenced articles included data beyond 2020, and analyses assessing the disruptive impact of the COVID‐19 pandemic on long‐term trends in the interventional therapy of AF are rare. Clinical associations between AF and COVID‐19 as well as prognostic implications of the co‐prevalence of the diseases have been described in detail, but data with regard to the treatment of AF patients irrespective of co‐existing viral infection is limited to the early phase of the COVID‐19 pandemic [38, 39, 40]. Up to September 2020, a markedly decrease in new diagnoses of AF has been noted in registries from both Europe and the USA, affecting both the in‐ and outpatient setting [41, 42]. Corresponding to the lower number of new AF diagnoses, the number of AF‐related inpatient admissions was reduced [12]. At the same time, a relevant percentage of AF patients awaiting CA refused to attend professional help despite having arrhythmia‐related symptoms [43]. Consequently, a substantial reduction of interventional therapies for AF were reported from different European countries in the first months of the pandemic. In an administrative database from the United Kingdom, AF CAs were reduced by 83% compared to data from 2019, which exceeded the downregulation of other cardiovascular procedures like pacemaker implantations or percutaneous coronary interventions [13]. Similar findings were reported in surveys of arrhythmia‐centers in Italy and among Chinese cardiologists [44, 45]. In line with our findings, three studies based on large, multicenter, administrative datasets from the United States confirmed an upward trend in the annual number of electrophysiological interventions during the second decade of the 2000s, followed by a decrease in case numbers in 2020, reaching a nadir in the second quarter of the year [46, 47, 48]. In contrast to these trends for electrophysiological interventions as a whole, one of these investigations showed that the number of pulmonary vein isolations actually increased further in 2020 compared to previous years [48]. Similarly, our data showed a reduction of hospital admissions for AF CA in 2020 with a subsequent delayed recovery and increase of case numbers above pre‐pandemic levels in 2022. The pandemic thus led to a shift in the trend curve to a temporarily lower level without affecting the trend as such. This is also in line with data from a national data platform from China that reported a decline of CA numbers in 2020 but an increase in 2021 compared to 2019 data [49]. Interestingly, this increase of case numbers in the later pandemic period was driven by a growth in secondary hospitals with a concomitant decline of procedures in centers with very high ablation volume. In our data, there was no interaction between CA case numbers and center volume. However, a lower number of procedures was observed in the center with the highest ablation volume when compared to the pre‐pandemic period.

### 4.1 Limitations

This study is based on administrative data that was not stored for research interests but for remuneration reasons, which potentially could affect the encoded information and harbors the risk of bias in the evaluation of various clinical endpoints. Quality of the results depends to a large extent on the correct encoding of procedures and diagnoses at hospital discharge. However, regarding the discharge diagnoses and the adequacy of hospitalization as well as encoding, there is a continuous evaluation by health insurances which supports the assumption of overall valid information. This also accounts for information presented in this study as it is relevant for reimbursement. It has been shown, that encoding accuracy of ICD‐10‐GM‐based first discharge diagnoses and OPS codes in administrative is good and reliable [50]. Due to the type of data, additional supporting information regarding patients’ specific medical history, imaging, laboratory results, medication and treatment‐related data was not available. The study relies on inpatient data from hospitals of a private German healthcare provider; therefore, generalizability of our data on the overall German or other healthcare systems may be discussed.

## 5 Conclusion

By analyzing a large, multicentric inpatient database, we showed an increase of AF CA procedures over the study period. Apart from a temporary dip in CA case numbers, the COVID‐19 pandemic had no sustained impact on this long‐term trend. There were relevant changes in the in‐hospital management and the procedural characteristics.

## Ethics Statement

There has been no public involvement associated with this study.

## Conflicts of Interest

All authors state that there is nothing to declare. All authors have completed the ICMJE uniform disclosure form (at http://www.icmje.org/disclosure-of-interest/) and declare: no support from any organization for the submitted work; no financial relationship to Biosense Webster Inc.; no financial relationships with any organizations that might have an interest in the submitted work in the previous 3 years; no other relationships or activities that could appear to have influenced the submitted work.

## Supporting information


Supporting File

## Data Availability

The data and computing codes underlying this article will be shared on reasonable request to the corresponding author.
