# Supplementary material for: Trend Analyses on Interventional Treatment of Atrial Fibrillation From 2016 to 2022: Insights From a Multicenter Hospital Database of Left Atrial Catheter Ablation Cases
Source: Clin Cardiol. 2026 Apr 27;49(5):e70280. doi: 10.1002/clc.70280 (PMC13112413; doi:10.1002/clc.70280)

**Supplemental Material**

Supplemental Table 1: ICD-codes used to calculate Elixhauser comorbidity index (weighting according to AHRQ algorithm)

Supplemental Table 2: ICD-codes used to calculate CHA_2_DS_2_-VASc-Score

Supplemental Table 3: Baseline characteristics comparing a pre-pandemic and a pandemic cohort overall and per center volume

Supplemental Figure 1: Participating center sorted by the total number of patients included

Supplemental Figure 2: Decomposition of additive time series in catheter ablation case numbers

**Supplemental Table 1: ICD-codes used to calculate Elixhauser comorbidity index**

| ICD*-codes used to calculate Elixhauser comorbidity index | | |
| --- | --- | --- |
| *Item* | *Weight* | *ICD*-10-GM codes* |
| AIDS / HIV | 0 | B20, B21, B22, B23, B24 |
| Alcohol Abuse | -1 | F10, E52, G62.1, I42.6, K29.2, K70.0, K70.3, K70.9, T51, Z50.2, Z71.4, Z72.1 |
| Blood Loss Anemia | -3 | D50.0 |
| Cardiac Arrhythmias | 0 | I44.1, I44.2, I44.3, I45.6, I47, I48, I49, R00.0, R00.1, R00.8, T82.1, Z45.00, Z45.01, Z95.0 |
| Chronic Pulmonary Disease | 3 | I27.8, I27.9, J40, J41, J42, J43, J44, J45, J46, J47, J60, J61, J62, J63, J64, J65, J66, J67, J68.4, J70.1, J70.3 |
| Chronic Renal Failure | 6 | I12.0, I31.1, N18, N19, N25.0, Z49.0, Z49.1, Z49.2, Z94.0, Z99.2 |
| Coagulopathy | 11 | D65, D66, D67, D68, D69.1, D69.3, D69.4, D69.5, D69.6 |
| Congestive Heart Failure | 9 | I09.0, I11.0, I13.0, I13.2, I25.5, I42.0, I42.1, I42.2, I42.5, I42.6, I42.7, I42.8, I42.9, I43, I50 |
| Deficiency Anemia | -2 | D50.8, D50.9, D51, D52, D53 |
| Depression | -5 | F20.4, F31.3 - F31.5, F32, F33, F34.1, F41.2, F43.2 |
| Diabetes Mellitus, Uncomplicated | 0 | E10.0, E10.1, E10.9, E11.0, E11.1, E11.9, E12.0, E12.1, E12.9, E13.0, E13.1, E13.9, E14.0, E14.1, E14.9 (excluding E10.2, E10.3, E10.4, E10.5, E10.6, E10.7, E10.8, E11.2, E11.3, E11.4, E11.5, E11.6, E11.7, E11.8, E12.2, E12.3, E12.4, E12.5, E12.6, E12.7, E12.8, E13.2, E13.3, E13.4, E13.5, E13.6, E13.7, E13.8, E14.2, E14.3, E14.4, E14.5, E14.6, E14.7, E14.8) |
| Diabetes Mellitus, Complicated | -3 | E10.2, E10.3, E10.4, E10.5, E10.6, E10.7, E10.8, E11.2, E11.3, E11.4, E11.5, E11.6, E11.7, E11.8, E12.2, E12.3, E12.4, E12.5, E12.6, E12.7, E12.8, E13.2, E13.3, E13.4, E13.5, E13.6, E13.7, E13.8, E14.2, E14.3, E14.4, E14.5, E14.6, E14.7, E14.8 |
| Drug Abuse | -7 | F11, F12, F13, F14, F15, F16, F18, F19, Z71.5, Z72.2 |
| Fluid And Electrolyte Disorders | 11 | E22.2, E86, E87 |
| Hypertension (combined uncomplicated and complicated) | -1 | I10, I11, I12, I13, I15 |
| Hypothyroidism | 0 | E00, E01, E02, E03, E89.0 |
| Liver Disease | 4 | B18, I85, I86.4, I98.2, K70, K71.1, K71.3, K71.4, K71.5, K71.7, K72, K73, K74, K76.0, K76.2, K76.9, Z94.4 |
| Lymphoma | 6 | C81, C82, C83, C84, C85, C88, C96, C90.0, C90.2 |
| Metastatic Cancer | 14 | C77, C78, C79, C80 |
| Neurological Disorders, other | 5 | G10, G11, G12, G13. G20, G21, G22, G25.4, G25.5, G31.2, G31.8, G31.9, G32, G35, G36, G37, G40, G41, G93.1, G93.4, R47.0, R56 |
| Obesity | -5 | E66 |
| Paralysis | 5 | G04.1, G11.4, G80.1, G80.2, G81, G82, G83.0, G83.1, G83.2, G83.3, G83.4, G83.9 |
| Peptic Ulcer Disease, Excluding Bleeding | 0 | K25.7, K25.9, K26.7, K26.9, K27.7, K27.9, K28.7, K28.9 |
| Peripheral Vascular Disorders | 3 | I70, I71, I73.1, I73.8, I73.9, I77.1, I79.0, I79.2, Z95.81, Z95.88, Z95.9 |
| Psychoses | -5 | F20, F22, F23, F24, F25, F28, F29, F30.2, F31.2, F31.5 |
| Pulmonary Circulation Disorder | 6 | I26, I27, I28.0, I28.8, I28.9 |
| Rheumatoid Arthritis / Collagen Vascular Diseases | 0 | L94.0, L94.1, L94.3, M05, M06, M08, M12.0, M12.3, M30, M31.0, M31.1, M31.2, M31.3, M32, M33, M34, M35, M45, M46.1, M46.8, M46.9 |
| Solid Tumor Without Metastases | 7 | C00, C01, C02, C03, C04, C05, C06, C07, C08, C09, C10, C11, C12, C13, C14, C15, C16, C17, C18, C19, C20, C21, C22, C23, C24, C25, C26, C30, C31, C32, C33, C34, C37, C38, C39, C40, C41, C43, C45, C46, C47, C48, C49, C50, C51, C52, C53, C54, C55, C56, C57, C58, C60, C61, C62, C63, C64, C65, C66, C67, C68, C69, C70, C71, C72, C73, C74, C75, C76, C97 |
| Valvular Heart Disease | 0 | I05, I06, I07, I08, I09.1, I34, I35, I36, I37, I38, I39, Q23.0, Q23.1, Q23.2, Q23.3, Z95.2, Z95.3, Z95.4 |
| Weight Loss | 9 | E40, E41, E42, E43, E44, E45, E46, R63.4, R64 |

* International Statistical Classification of Diseases and Related Health Problems (ICD-10-GM [German Modification]

**Supplemental Table 2: ICD-codes used to calculate CHA_2_DS_2_-VASc-Score**

| ICD*-COdes used to calculate CHA_2_DS_2_-VAsc-Score | | |
| --- | --- | --- |
| *Condition* | *Weight* | *ICD*-10-GM codes* |
| Congestive heart failure | 1 | I11.0, I13.0, I13.2, I25.5, I42.0, I42.1, I42.2, I42.5, I42.6, I42.7, I42.8, I42.9, I43.0, I43.1, I43.2, I43.8, I50.0, I50.1, I50.9 |
| Hypertension | 1 | I10.x, I11.x, I12.x, I13.x, I15.x, I67.4 |
| Age ≥75 years | 2 | N/A^#^ |
| Diabetes mellitus | 1 | E10.x, E11.x, E12.x, E13.x, E14.x, E15 |
| Previous stroke / TIA | 2 | I61.x, I63.x, I64.x, I69.x |
| Vascular disease | 1 | I25.x, I65.x, I66.x, I67.9, I70.x, I74.x |
| Age 65-74 years | 1 | N/A^#^ |
| Sex category (female) | 1 | N/A^#^ |

* International Statistical Classification of Diseases and Related Health Problems (ICD-10-GM [German Modification],

# Not applicable (data directly extracted from administrative data)

All ICD-10-GM codes ending on “.x” are meant as coding groups that include all corresponding sub-codes.

**Supplemental Table 3: Baseline characteristics comparing a pre-pandemic and a pandemic cohort overall and per center volume**

| Variable | Total cohort | | | Low-volume center | | | High-volume center | | |
| --- | --- | --- | --- | --- | --- | --- | --- | --- | --- |
|  | Pre-pandemic period | Pandemic period | P value | Pre-pandemic period | Pandemic period | P value | Pre-pandemic period | Pandemic period | P value |
| Number of cases | 15,880 | 13,264 | / | 1,686 | 1,407 | / | 14,194 | 11,857 | / |
| Type of hospital admission: urgent [%] | 17.9 | 17.5 | 0.42 | 23.5 | 28.0 | < 0.01 | 17.2 | 16.3 | 0.05 |
| Age, mean [years] | 64.8±10.4 | 66.0±10.1 | <0.001 | 66.4±9.8 | 66.8±10.0 | 0.3 | 64.6±10.5 | 66.0±10.1 | < 0.001 |
| Female sex [%] | 40 | 41 | 0.064 | 43 | 44 | 0.6 | 40 | 41 | 0.077 |
| CHA_2_DS_2_-VASc-Score | 2.3±1.6 | 2.4±1.6 | 0.001 | 2.9±1.6 | 2.9±1.7 | 0.6 | 2.2±1.5 | 2.3±1.6 | <0.001 |
| ECI, mean ± SD | 3.1±6.9 | 3.3±6.8 | 0.029 | 5.7±7.5 | 5.8±7.6 | 0.7 | 2.8±6.8 | 3.0±6.6 | 0.026 |

ECI: Elixhauser comorbidity index; SD: standard deviation

**Supplemental Figure 1: Participating center sorted by the total number of patients contributed**


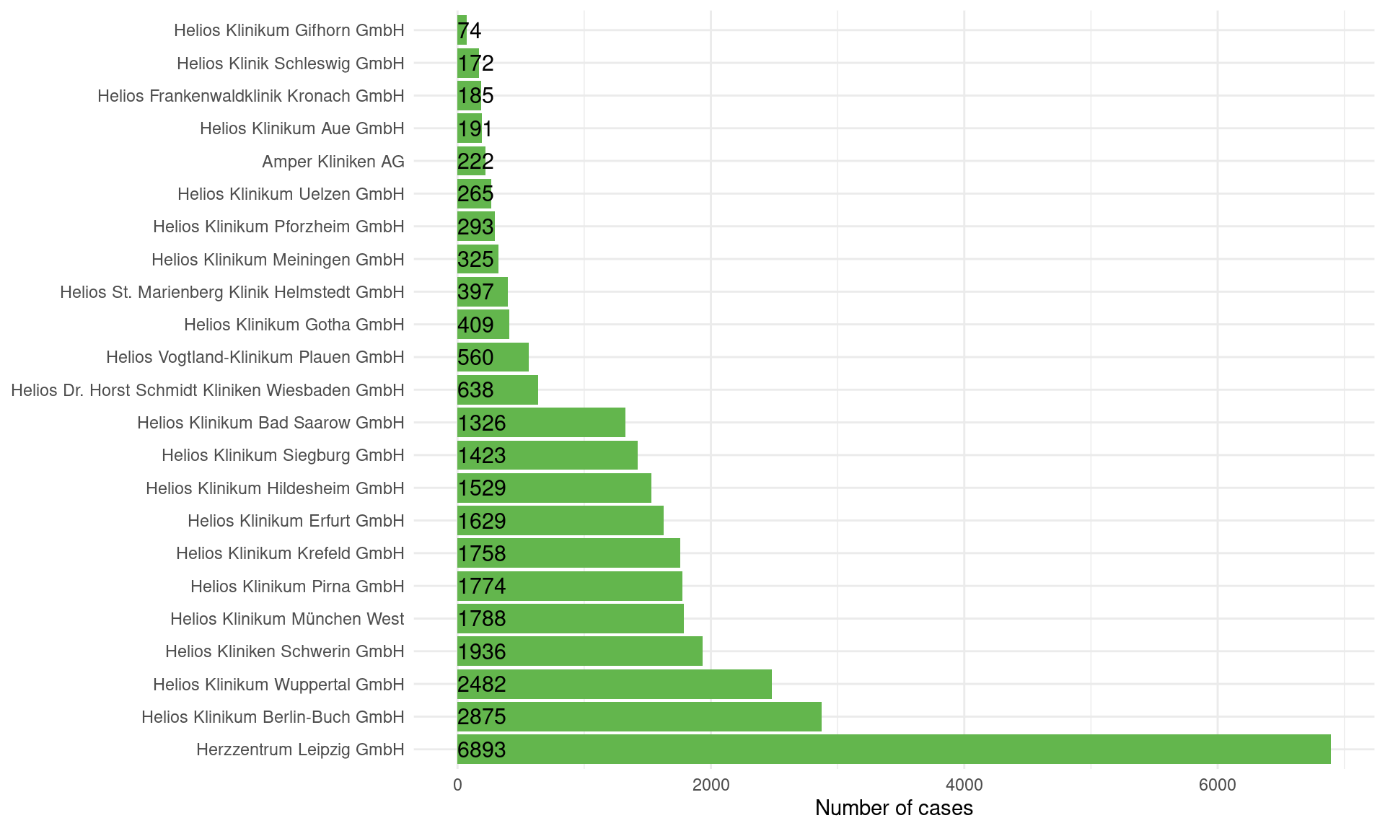


**Supplemental Figure 2: Decomposition of additive time series in catheter ablation case numbers**


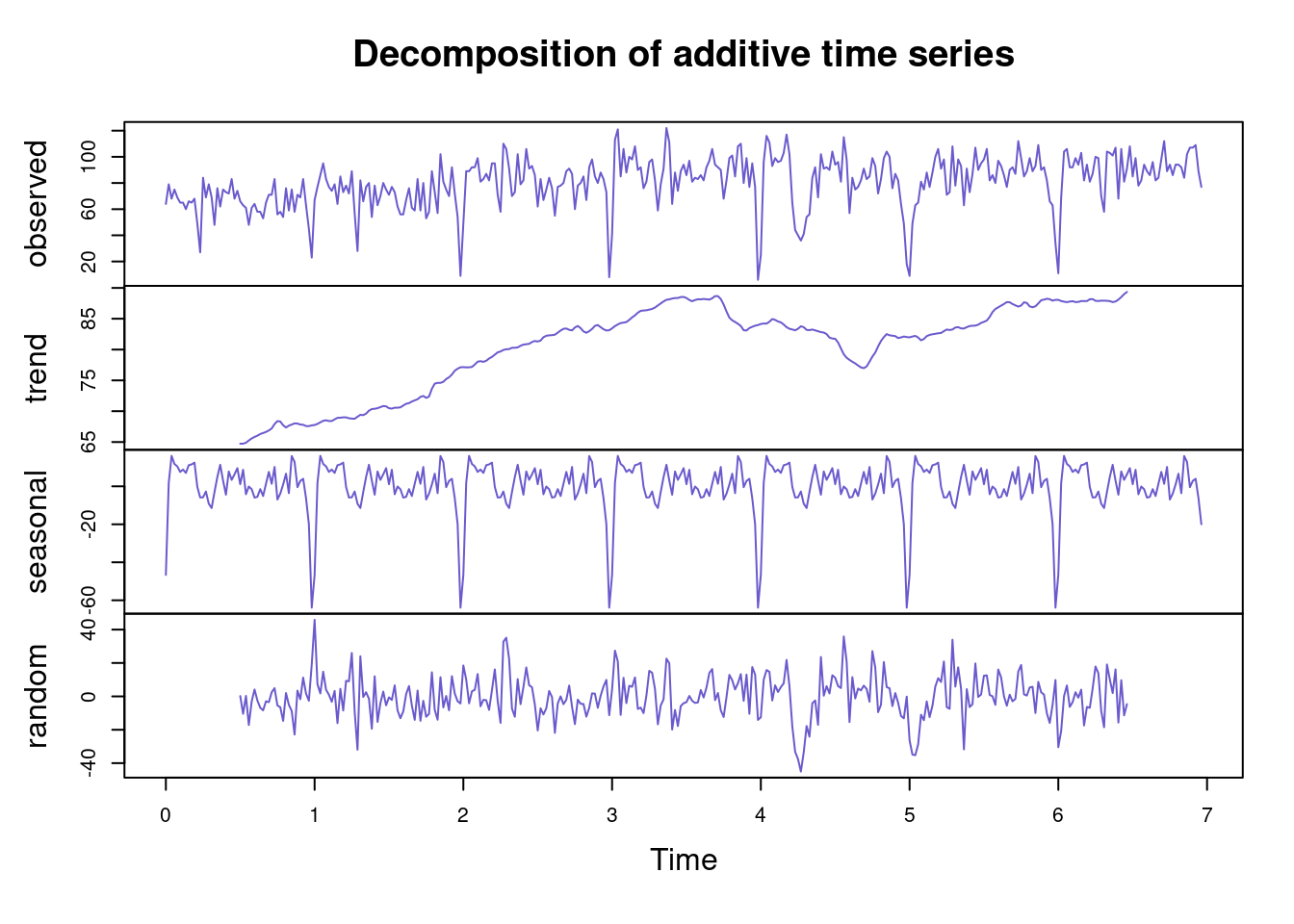

Supplement: Supplementary file 1 — Supporting File [file CLC-49-e70280-s001.docx]
